# Alcohol and Smoking Mediated Modulations in Adaptive Immunity in Pancreatitis

**DOI:** 10.3390/cells9081880

**Published:** 2020-08-11

**Authors:** Rakesh Bhatia, Christopher Thompson, Koelina Ganguly, Shailender Singh, Surinder K. Batra, Sushil Kumar

**Affiliations:** 1 Department of Biochemistry and Molecular Biology, University of Nebraska Medical Center, Omaha, NE 68198-5870, USA; rocky.bhatia@unmc.edu (R.B.); c.thompson@unmc.edu (C.T.); koelina.ganguly@unmc.edu (K.G.); 2 Division of Gastroenterology-Hepatology, Department of Internal Medicine, University of Nebraska Medical Center, Omaha, NE 68198-5870, USA; shailender.singh@unmc.edu; 3 Fred and Pamela Buffett Cancer Center; University of Nebraska Medical Center, Omaha, NE 68198-5870, USA; 4 Eppley Institute for Research in Cancer and Allied Diseases, University of Nebraska Medical Center, Omaha, NE 68198-5870, USA

**Keywords:** pancreatitis, adaptive immunity, alcohol, smoking, acute pancreatitis, chronic pancreatitis, lymphocytes, pancreatic stellate cells, collagen

## Abstract

Pancreatitis is a condition of pancreatic inflammation driven by injury to the pancreatic parenchyma. The extent of acinar insult, intensity, and type of immune response determines the severity of the disease. Smoking, alcohol and autoimmune pancreatitis are some of the predominant risk factors that increase the risk of pancreatitis by differentially influencing the adaptive immune system. The overall decrease in peripheral lymphocyte (T-, B- and (natural killer T-) NKT-cell) count and increased infiltration into the damaged pancreatic tissue highlight the contribution of adaptive immunity in the disease pathology. Smoking and alcohol modulate the responsiveness and apoptosis of T- and B-cells during pancreatic insult. Acute pancreatitis worsens with smoking and alcohol, leading to the development of systemic inflammatory response syndrome and compensatory anti-inflammatory response syndrome, suggesting the critical role of adaptive immunity in fatal outcomes such as multiple organ dysfunction. The presence of CD4^+^ and CD8^+^ T-lymphocytes and perforin-expressing cells in the fibrotic tissue in chronic pancreatitis modulate the severity of the disease. Due to their important role in altering the severity of the disease, attempts to target adaptive immune mediators will be critical for the development of novel therapeutic interventions.

## 1. Introduction

The pancreas is a unique organ due to the presence of its exocrine and endocrine compartments. The pancreatic acini perform an exocrine function by producing proteolytic enzymes as inactive precursors, which are activated in the intestinal lumen. The premature activation of these proteolytic enzymes in the pancreas, predominantly due to dysfunctional calcium homeostasis, leads to pancreatic autodigestion, which elicits an acute local inflammatory response, termed acute pancreatitis (AP). The release of pro-inflammatory cytokines by injured acini leads to leukocyte infiltration, which further releases a gamut of inflammatory mediators that aggravate tissue injury, as well as local and systemic inflammatory responses. The common etiological factors for acute pancreatitis include alcohol, smoking, gallstones, autoimmunity and genetic susceptibility. These etiological factors predispose the pancreas to recurrent AP (RAP), resulting in activation of pancreatic stellate cells (PSC), which leads to the displacement of pancreatic parenchyma with extensive fibrosis and extracellular matrix (ECM) proteins, a condition known as chronic pancreatitis (CP).

Sarles et al. first demonstrated the involvement of immune-mediated mechanisms in pancreatitis pathology [1]. An initial event during AP is the recruitment of neutrophils, which are otherwise untraceable in the normal pancreas [2]. Subsequently, there is recruitment of other immune cells from the innate and adaptive arm, such as monocytes, dendritic cells (DCs), T- and B-lymphocytes, as well as platelets. The presence of nicotinamide adenine dinucleotide phosphate (NADPH) oxidase in infiltrating neutrophils ameliorates oxidative stress, contributing to trypsin activation and increased damage to the pancreatic acinar cells [3]. Chemokines, such as CCL2, CCL3 and CCL5, released from the damaged acinar cells, lead to the recruitment of monocytes [2,4], and activated monocytes further amplify the inflammatory response by increasing the production of TNF-α, IL-1 and IL-6, promoting disease progression [5]. Macrophages are the major source of IL-6, which is differentially regulated in cerulein-induced murine models of pancreatitis and is associated with acute injury [6,7]. Akin monocytes and macrophages, DCs, also serve as a warehouse for various pro-inflammatory mediators of acinar cell damage. However, DCs have been shown to play a dichotomous role in AP due to their ability to promote or suppress the inflammatory response [8,9]. Studies have demonstrated the involvement of DCs in restraining the disease, observing that systemic depletion of DCs leads to severe acinar cell damage, increased pancreatic dysfunction and mortality [10]. DCs have also been shown to contribute significantly to the pathology of CP, by modulating the adaptive immune system. Therefore, both innate and adaptive immune arms have a significant role in the initiation of pancreatitis and its severity, as well as in multiple organ failure (MOF). The participation of innate immune mediators such as neutrophils, monocytes and DCs in modulating the severity of AP has been discussed elsewhere [8,11,12,13]. However, the selective contribution of the adaptive immune arm, i.e., T- and B-lymphocytes, in modulating disease severity during acute and chronic pancreatitis has scarcely been reviewed. Therefore, this review highlights the role of the adaptive immune response and environmental factors like smoking and alcohol in influencing and orchestrating the pathology and severity of acute and chronic pancreatitis.

## 2. Role of Adaptive Immune Mediators in Pancreatitis

**Figure 1:**
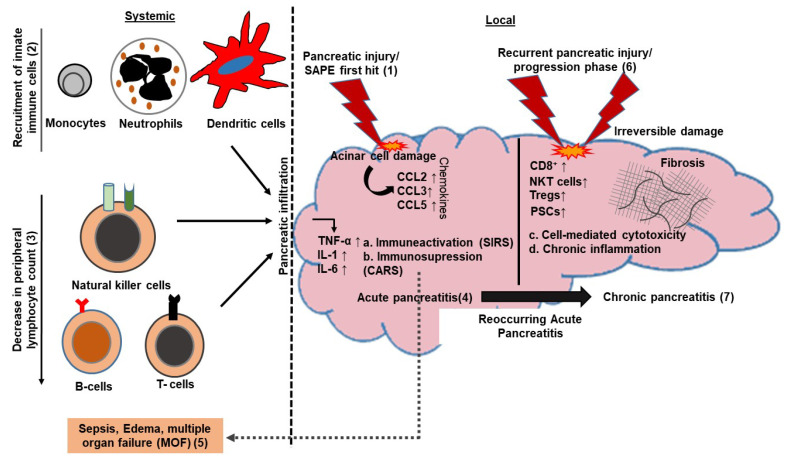
Sequential events involving adaptive immune mediators in clinical progression of the disease. Initial pancreatic acinar cell damage (1) by either trypsin autodigestion or any other insult release various chemokines, resulting in the recruitment of innate immune effectors (2) at the site of injury/damage. This follows the increased lymphocyte infiltration into the pancreas, resulting in a significant reduction of peripheral lymphocyte count (3). Infiltrating immune cells lead to the release of various pro-inflammatory cytokines, resulting in immune hyper-activation (a) due to uncontrolled local inflammatory response. Initial immune-hyper-activation is followed by a phase of immunosuppression (b), leading to a compensatory anti-inflammatory response, resulting in sepsis and edema due to secondary infections and, ultimately, multiple organ failure (MOF) (5). Chronic pancreatitis (CP) (7) is a state of chronic inflammatory response (d) caused by recurrent pancreatic injury (6), leading to irreversible damage, an increased cytotoxic cell (c) infiltration and activation of pancreatic stellate cells, promoting necrosis and fibrosis. Smoking and alcohol are among the major contributing factors for the progression of acute pancreatitis (AP) (4) to CP (7). SAPE; sentinel acute pancreatitis event. CARS; compensatory anti-inflammatory response syndrome.

The severity of AP depends upon the balance between the pro- and anti-inflammatory responses during disease progression [9]. The contribution of adaptive immune mediators in pancreatitis pathology is demonstrated in athymic or mice deficient in CD4^+^ and CD8^+^ T-cells [14]. That study suggested the role of CD4^+^ T-cells in tissue injury during AP. Furthermore, an increased lymphocyte flux into the injured pancreas and an overall decrease in peripheral B- and T-cell numbers have been observed in AP. This decrease in lymphocyte count is further aggravated as the severity of disease worsens. Markedly high levels of infiltrating cytolytic lymphocytes, such as CD8^+^ T-cells, natural killer (NK) cells and NKT-cells have also been observed in pancreatic tissues of CP patients. CP patients have higher IL-10-producing Foxp3^+^ regulatory T-cells, which suppresses the proliferation of autologous T-cells in an antigen-specific manner [15]. The overall intrapancreatic increase in regulatory T- and B-cell numbers during AP onset is the predictor of MOF and the severity of AP. Figure 1 shows the crosstalk between immune mediators (adaptive and innate) in aggravating disease severity in AP and CP.

### 2.1. Adaptive Immunity in Acute Pancreatitis

The infiltrating lymphocytes play an important role in AP. Their genetic ablation in AP mouse models shows reduced disease severity compared to wild-type controls [14,16,17]. Initial AP episodes are characterized by immunosuppressive events with overall T- and B-lymphocyte impairment [17,18,19], followed by a decrease in T- and B-lymphocytes in circulation, associated with the severity of the disease. Among T-lymphocytes, CD4^+^ T-cells peripherally deplete to a greater extent than CD8^+^ T-cells in both moderate acute pancreatitis (MAP) and severe acute pancreatitis (SAP), due to their increased infiltration from blood vessels to the site of injury bordering acini [14]. The increased T-cell infiltration into the inflamed pancreas is due to the systemic increase in IL-6 and CXCR2-ligands from the acinar cells and increased pro-inflammatory cytokine production from innate immune cells during AP [9,20]. During MAP, as the disease resolves, the CD4^+^ T-cell number returns to the normal range; however, the number remains low if the disease progresses to SAP, characterized by necrosis and tissue abscess [17,21]. The overall decrease in T helper cell count has been attributed to pancreatic necrosis and the presence of injury-induced endotoxins in circulation from the gastrointestinal tract [18]. However, the CD8^+^ T-cell population remains unaltered. Of note, an increase in AP pathology by infiltrating CD4^+^ T-cells is not mediated through their cross-activation by antigen-presenting cells (APCs); however, the T-cell mediated cytotoxicity is driven by Fas–Fas ligand activation, as confirmed by increased Fas ligand mRNA expression following cerulein injections, resulting in increased necrosis [14].

Apart from the pancreatic infiltration, the overall reduction in T-lymphocytes in AP is further supported by decreased levels of T-cell survival factor, IL-2, in mononuclear splenic cells in the AP murine model [22]. High expression of CD95/Fas-R/Apo-1 receptor proteins and annexin-V staining also suggests T-cell apoptosis as the major reason for decreased CD4^+^ T-lymphocytes during the early course of disease [17]. The onset of AP may lead to systemic immunological response syndrome (SIRS), MOF or mortality, depending upon the disease severity [23]. Activated lymphocytes (T- and B-cells) are central to the onset of the SIRS response. Initially, the progression of AP towards SIRS is due to immune hyper-activation caused by an uncontrolled local inflammatory response, leading towards the excessive systemic activation of peripheral inflammatory cells. As SIRS progresses, increased intracellular activation of caspases leads to the induction of apoptosis of circulating CD4^+^ T-lymphocytes and increased NK cell activation [24]. However, inflammatory conditions persisting for more than two weeks leads to compensatory anti-inflammatory response syndrome (CARS), creating an immunosuppressive environment, leading to increased susceptibility of necrotic tissue to infections by intestinal flora and secondary MOF [25,26,27].

According to the revised Atlanta classification, 2012 [28], if the organ failure persists for more than 48 h, it is categorized as persistent organ failure (POF), otherwise known as transient organ failure (TOF). Recent studies demonstrated altered T- and B-lymphocyte levels as a predictor of organ failure in AP [29,30]. There is a significant increase in B-cell count in patients with OF, in contrast to the lower CD4^+^ T-cell numbers. Importantly, the decrease in CD4^+^ T-cell numbers was significant across all etiologies; however, the increase in B-lymphocyte count was only significant in gallstone-mediated AP [30]. A decrease in CD4^+^ T-cell count, resulting in increased immunosuppression, is speculated to be one of the causes of OF [30]. In addition, a study on 39 patients with POF and 30 patients with TOF demonstrated a significant reduction in the total number of peripheral CD4^+^ T-cells in the patients with POF, suggesting its utility as a potential indicator for POF during AP [29].

Studies on murine models of experimental pancreatitis also demonstrated that T-lymphocytes influence the innate immune response. CD4^+^ T-lymphocytes, after infiltrating into the pancreas, are involved in promoting macrophage activation and Fas–Fas ligand-mediated cytotoxicity during the early onset of AP [21]. A decrease in the number of IL-22^+^ CD4^+^ T-cells during AP leads to increased disease aggressiveness. The IL-22 receptor A1 (IL-22RA1) is expressed on acinar cells responding to IL-22, which protects the acinar cell from apoptosis by activating signal transducers and activators of transcription 3 (STAT3) signaling [31]. Altogether, the early detection of Th1 cytokines in the blood is a predictor of the severity of AP in patients. The course and magnitude of T- and B-cell depletion are similar in MAP; however, a comparatively higher B-lymphocyte reduction is observed in SAP, as shown by a shift in the CD7^+^/CD19^+^ ratio [17]. A decrease in regulatory B-cells (B-regs) is also associated with increased severity of AP. A study showed a decrease in CD19^+^CD24^hi^CD27^hi^ memory B-cells and IL-10-producing B-cells in patients with SAP. In addition, CD19^+^CD24^hi^CD27^hi^ memory B-cells also suppressed cytokine production from CD4^+^ T-cells and CD14^+^ monocytes in AP. Usually, B-regs release an abundant amount of IL-10 and TGFβ, leading to immunosuppression and induction of T-reg populations. However, in AP, these cells showed abnormal functions and were implicated in the reduction of cytokine production from CD4^+^ T-cells, inducing an impaired CD4^+^ T-cell response [32]. The precise mechanism by which these B-lymphocytes perform different functions remains unaddressed. Taken together, the apoptotic environment created by infiltrating T- and B-lymphocytes during the disease onset leads to exposure to pancreas-specific antigens, generating an acute inflammatory response and increased severity in AP.

### 2.2. Adaptive Immunity in Chronic Pancreatitis

Unlike AP histology with prominent necrosis and inflammation of pancreatic acini, CP is a persistent pancreatic inflammatory syndrome encompassing tissue fibrosis, deposition of ECM proteins and acinar cell atrophy. Patients with CP have a higher risk of getting pancreatic cancer, and the risk is even higher in patients with hereditary pancreatitis [12,33,34]. Unlike AP, CP leads to irreversible damage to the pancreas, leading to gross pancreatic exocrine and endocrine insufficiencies. Clinically, 4–24% of patients have been reported to progress from recurrent AP to CP, and the incidence is higher in alcohol and tobacco users. This is further supported by the fact that 78% of patients progressed to CP from recurrent AP who continued to consume alcohol [11]. Compared to the normal pancreas, increased lymphocyte numbers are observed in pancreatic tissue in CP. The CD8^+^ T-cells are considered to be predominant contributors to disease severity during CP, populating and residing between the pancreatic parenchyma and the fibrotic area. CD8^+^ T-cells expressing CD103 (αE-antigen), analogous to intestinal epithelial lymphocytes, are also present between ductal cells in CP [35], suggesting the protective role of these cells against the damaging effects of epithelial cells. Cell-mediated cytotoxicity by CD8^+^ T-cells or NKT cells is hypothesized to be the major player in CP pathology [35,36]. The infiltration of perforin-expressing CD8^+^ T-cells and, to some extent, CD56^+^ NKT cells [36], abundance of CD8^+^ memory T-cell subsets [15] and the association of CD8^+^ T-cells with pancreatic acinar cells and fibrotic tissue [37] establishes the involvement of the cytotoxic immune response in CP. Increased CD8^+^ T-cell infiltration and presence is observed in interlobular connective tissue and parenchyma in the CP model of male Wistar rats with increasing disease severity. The CD8^+^ T-cells come in close association with acinar cells, leading to their apoptosis and replacement by fibrotic compartments [37]. Treatment of mice with T-cell suppressant Tacrolimus rescued acinar cell apoptosis, suggesting an association between T-cells and acinar cells during the development of CP. CP involves a cascade of inflammatory and suppressive events, as demonstrated by the increased number of IL-10^+^IFNγ^−^FoxP3^+^ T-regs against pancreatitis-associated antigens, counteracting the damage caused by cytotoxic cells in CP [15]. In addition, the restoration of the immunosuppressive environment with increased T-reg cells and the elimination of chronic inflammatory focus upon pancreatic head resection in CP patients have established the role of infiltrating CD3^+^ T-cells in CP pathology [38].

There is an increase in intracellular reactive oxygen species (ROS) associated with CP progression and fibrogenesis. In the L-arginine based CP model, Chen et al. tested the therapeutic potential of hydrogen treatment as a potent scavenger of hydroxyl radicals. There was an overall improvement in CP pathology upon hydrogen treatment, as L-arginine-induced pancreatitis leads to ROS–mediated T-lymphocyte apoptosis, especially T-regs, and restoration of depleted T-regs by hydrogen treatment decreased disease severity and promoted their survival [39]. Apart from the pancreatitis-associated antigens and IL-10-producing FoxP3^+^ T-regs, CP patients have an increased number of antigen-specific CCR7^+^CD45RA^−^ central memory T-cells as compared to the healthy and CP-resected controls [40]. T-cells derived from CP patients responded towards pancreatitis-associated antigens, increasing IL-10-based responses; however, the T-cells obtained from pancreatic cancer patients responded towards pancreatic cancer-associated antigens, resulting in high IFN-γ levels. This shows that CP patients have more of an immunosuppressive environment, involving higher T-reg activity [11]. Unlike FoxP3^+^ T-reg cells, which are involved in suppressive function, increased memory T-cells are implicated in maintaining the inflammatory environment in CP. The CP patients have a 16-fold higher chance of progression towards pancreatic carcinoma, and Th2 cells are among the major contributors to this. Th2 cells also contribute to pancreatic inflammation, and the activated DCs are responsible for skewing T-cell differentiation into the Th2 phenotype upon MyD88 inhibition. This activation of DCs by the MyD88 blockade, resulting in the Th2 phenotype, leads to increased inflammation and fibrosis during CP and promotes ductal transformation [41]. Along with lymphocytes, pancreatic stellate cells (PSCs) promote CP pathology by promoting tissue fibrosis. PSCs are activated during CP by a number of toxic and immune-modulatory factors, such as ethanol, smoking, cytokines and chemokines, leading to either synthesis or degradation of ECM proteins. These activated stellate cells, together with T- and B-lymphocytes, increase the inflammatory response and ECM deposition in the pancreas as the CP becomes more severe.

## 3. Smoking and Alcohol-Mediated Modulation of Adaptive Immunity in Pancreatitis

Smoking and alcohol are among the common epidemiologically established risk factors for pancreatitis. Studies show that smokers and alcoholic patients exhibit a higher risk of recurrent AP, leading to the progression of CP. Smoking synergizes the effect of alcohol toxicity and predisposition towards pancreatic disorders. For instance, more than 80% of patients with alcohol-induced chronic pancreatitis are smokers or use tobacco [42]. Overall, smoking and alcohol show profound effects on humoral or adaptive immune effector function. Continuous smoking and heavy alcohol consumption act as a metabolic stress factor to the immune system, which in turn leads to an altered immune response, resulting in increased anti-inflammatory cytokine production and fibrosis [43]. The synergism between alcohol and smoking in promoting pancreatitis follows the “multiple hits on multiple targets” model, which involves crosstalk between multiple pathways, leading to exacerbated acinar damage during pancreatitis. At the cellular level, alcohol and smoking together increase ER stress, elevate intracellular calcium levels, promote mitochondrial damage, increase oxidative stress, activate PSCs and increase pancreatic fibrosis. Among these pathways, Lugea et al. showed that smoking and alcohol predominantly induce an unresolved ER stress response and amplify the risk and severity of pancreatitis [44,45]. Persistent smoking and incidence of CP follow a dose-response curve [46]. The major complication of prolonged smoking is the calcification of pancreata and exocrine insufficiency [47].

Smoking significantly alters T-(CD4^+^ and CD8^+^) and B-lymphocyte function through alpha 4 and alpha 7 nicotine acetylcholine receptors (α4/α7nAChR) [48,49]. Upon nicotine exposure, these receptors induce Th17^+^ T-cell-mediated inflammation and auto-reactive B-cells, resulting in asthma or other atopic diseases [50]. Chronic smoke exposure (CSE) alters overall T-cell responsiveness by decreasing cell proliferation, inhibiting antibody forming B-cells and inducing T-cell anergy. This effect is mediated by the depletion of intracellular Ca^2+^ stores, which accounts for the overall loss of T-cell function and aberrant signaling [51]. CD8^+^ T-cell knockout mice were unable to respond to CSE as CD8^+^ cells secrete IP-10 upon CSE, which in turn activates macrophage elastase, resulting in elastin fragmentation and injury [52]. CD4^+^ T cells are the major source of IL-22 in the pancreas [31]. Cigarette smoke contains different AhR (aryl hydrocarbon receptor) ligands, such as dioxin and benzo (a) pyrene (BaP) [53,54]. AhR activation by these ligands leads to increased IL-22 production by CD4^+^ T-cells, which in turn promotes tissue fibrosis by PSC activation and ECM deposition [47,55]. Mechanistically, components of cigarette smoke, such as nicotine and NNK, activates AhR signaling and promotes the expression of IL-22 in CD4^+^ T-cells. The IL-22 receptor (IL22RA1) present on the surface of PSCs actively responds to IL-22 secreted from T-lymphocytes, leading to phosphorylation of STAT3, which then translocates into the nucleus, resulting in upregulation of ECM proteins such as collagen 1A1 (COL1A1) and fibronectin 1 (FN1). This increase in ECM proteins leads to extensive fibrosis and progressive degeneration of normal pancreatic parenchyma [47]. However, that study shows another interesting finding, in which the PSCs expressing COL1A1 and FN1 upon IL-22 activation failed to express canonical activation markers such as alpha smooth muscle actin (αSMA) and transforming growth factor-β (TGFβ). Collectively, these reports establish the influence of smoking on adaptive immune mediators exacerbating (the complications of) CP pathology. Apart from T-cells, it is also expressed on pancreatic acinar cells and is regarded as a regulator of metabolic processes.

Similarly, a reduction in the number of CD4^+^ and CD8^+^ T-cells in heavy drinkers [56,57] and chronic ethanol-fed mice [58,59,60] demonstrates the negative effect of alcohol on adaptive immune function during AP. Long-term alcohol exposure alters the naïve T-cell differentiation profile, leading to the formation of the memory phenotype. However, chronic alcohol consumption, both in humans and mice, results in increased activation of CD8^+^ T-cells, which could contribute to the induction of alcohol-induced CP [60]. Increased accumulation of memory T-cells with a concomitant decrease in naïve T-cells is associated with the onset of different inflammatory and age-related diseases [61,62]. In addition to the total lymphocyte count, alcohol also alters the T-lymphocyte phenotype. Chronic alcohol exposure leads to increased T-cell activation-induced cell death, differentiates T-cells towards the memory phenotype and results in altered thymocyte development. These factors further affect B-cell development and maturation, resulting in increased levels of autoreactive antibodies [60], contributing to CP pathology. In addition, increased IgA and IgM levels both in heavy drinkers and mouse hybridomas treated with ethanol demonstrate the altered B-cell response, upon alcohol exposure [56,63]. The reason behind B-cell activation by alcohol is protein adducts and membrane lipid peroxidation by acetaldehyde, leading to an increased immunogenicity and autoimmune response [64]. Alcohol alone is not able to cause AP but acts as a metabolic stress factor, lowering the threshold of trypsin activation and increasing the susceptibility of pancreatic inflammation in the presence of other risk factors [45,65,66]. Furthermore, alcohol promotes anti-inflammatory effects by enhancing IL-10 release and inhibiting tumor necrosis factor-alpha (TNF-α) production. However, its prolonged exposure increases gut permeability, resulting in the release of endotoxins and lipopolysaccharides (LPSs), leading to pancreatic cell necrosis during alcoholic CP [67,68]. In addition, necrotic cell death caused by alcohol during AP is a strong activator of the immune response and increases the severity of AP [46].

**Figure 2:**
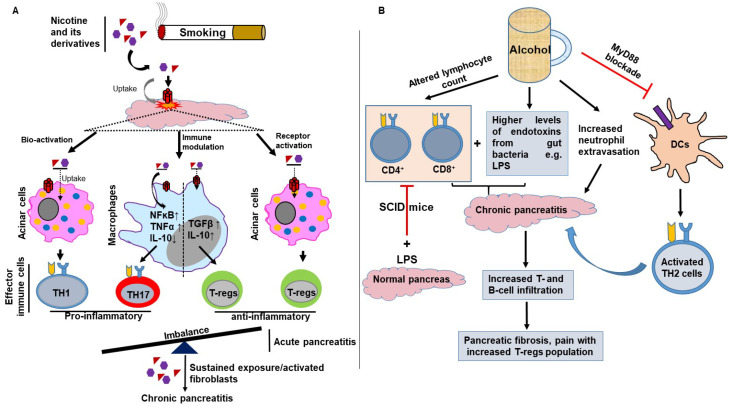
Adaptive immunity modulates smoke and alcohol-induced severity of AP. (**A**) Smoking contains nicotine and its derivatives. These smoking byproducts interact with their receptors, present on acinar and stellate cells, leading to the release of various chemokines, resulting in infiltration as well as activation of T- and B-lymphocytes, resulting in pancreatic inflammation. (**B**) Alcohol, on the other hand, negatively affects the adaptive immune function (CD4^+^ and CD8^+^ T cell count) in AP. Unlike smoking, alcohol cannot independently induce pancreatitis but creates a predisposition towards the disease’s progression by increasing susceptibility, in conjunction with the other risk factors. During CP, the pancreas is infiltrated by T- and B-cells, there is release of endotoxins and inflammatory components such as lipopolysaccharides (LPSs) due to alcohol-induced gut permeability, which further aggravates the disease pathology, resulting in pancreatic necrosis and fibrosis.

Multiple studies have exploited murine models to demonstrate the effects of alcohol in promoting pancreatic fibrosis in alcoholics and alcohol-induced acinar cell stress and altered immune function [69,70,71]. A study using an animal model for alcoholic chronic pancreatitis explains how alcohol promotes an anti-inflammatory environment during the recovery phase. After three consecutive AP episodes in alcohol-fed mice, there was an increased expression of anti-inflammatory mediators such as TGFB1, HIF1A and HIF3A. This overall anti-inflammatory stimulus promotes the tissue-specific expression of COL1A1, COL1A2 and FN1 in alcohol-fed mice, promoting disease in a pro-fibrotic direction. This was demonstrated by the striking differences in the pancreatic histology of mice subjected to AP either alone or in combination with chronic alcohol feeding. In comparison to the normal histology of pancreata from control (non-alcohol-fed) mice, the chronic alcohol-fed AP mice showed prominent necrotic areas with extensive fibrosis and calcification [69]. This model closely resembles the disease pathology observed in CP patients with heavy drinking history. Collectively, chronic alcohol exposure leads to pancreatic acinar cell stress, resulting in the release of stress-induced cytokines, which promote an anti-inflammatory environment. These anti-inflammatory mediators, such as IL-10 and TGFB1-producing T-cells and innate immune cells activate PSCs and fibroblasts, resulting in extensive fibrosis, a hallmark of CP. Unlike alcoholic and obstructive pancreatitis pathology, which exhibits inflamed and necrotic tissue, non-alcoholic duct destructive CP (NADDCP) shows ductal epithelial alterations, periductal-inflammation and fibrosis, nearly prompting the suspicion of pancreatic cancer. The major difference between alcoholic CP and NADDCP includes the diffused topography of lesions, the absence of calcification and cysts and the presence of mast cells, creating an inflammatory environment. The involvement of smoking and alcohol in shifting the inflammatory response from AP to chronic pancreatic inflammation, and the role of different molecular mediators, is shown in Figure 2.

PSCs are the key players in the fibrogenesis of CP. PSCs undergo hyper-proliferation and transformation to an activated state, resulting in the deposition of ECM components. Several studies have elucidated the role of ROS and soluble mediators from infiltrating immune cells in the activation of PSCs; however, few recent studies have shown the direct activation of PSCs upon exposure to cigarette smoke and alcohol. Like T- and B-cells, PSCs express nicotine acetylcholine receptors (nAChRs), which make them susceptible to activation by CSE. In the same study, it was found that nicotine-derived nitrosamine ketone (NNK), an active ingredient of CSE, either alone or in combination with alcohol, activates the α7nAChRs on human PSCs, leading to an increase in their proliferation, migration and collagen-I synthesis [72]. A recent study demonstrated that proliferation and α-SMA expression by human PSCs significantly increased upon exposure to nicotine. The nicotine-induced activation of human PSCs was associated with α7nAChR/Jak2-STAT3 axis-mediated autophagic induction [73]. Considering the heterogeneous cell types, defined spatial distribution and closely interactive physiology of the cellular components of the pancreas, it is tempting to speculate that in the face of an inflamed milieu and disruption of tissue architecture, there may be profound crosstalk between the injured acini, PSCs and the infiltrating immune cells. Indeed, a recent study demonstrated that upon alcohol-induced injury to the acini, proteases like kallikrein are released, which act on kininogens to produce bradykinin, that in turn results in activation of PSCs via Ca2+ signaling. This phenomenon can be further aggravated by the combination of alcohol and fatty acid ethyl esters (FAEEs) or bile acids that further lead to acini degeneration and the release of kallikrein via Ca2+ signaling [74].

## 4. Other Factors Influencing Adaptive Immunity in Pancreatitis

In addition to smoking and alcohol, autoimmune pancreatitis, gallstones and genetic predisposition are the other common etiological factors that influence the incidence and severity of pancreatitis. All these factors show a differential impact on adaptive immune effector function driving disease severity. According to international consensus diagnostics criteria (ICDC), 2011 [75], autoimmune pancreatitis (AIP) is categorized into two major types, i.e., type I AIP and type II AIP. Type I AIP, also called lymphoplasmacytic sclerosing pancreatitis (LPSP), is characterized by increased IgG4 serum levels, whereas type II AIP, also known as idiopathic duct-centric pancreatitis (IDCP) includes the presence of granulocytic epithelial lesions (GEL) and the absence of IgG4 antibodies [75,76]. Narrowing of pancreatic ducts, enlargement of the pancreas and the presence of CD4^+^ and CD8^+^ T-cells and IgG4-bearing plasma B-cells are among the major differentiating factors of AIP from AP. The presence of autoantibodies increased infiltration of TLR-7-positive M2 macrophages in pancreatic tissues, and an increase in B- and T-lymphocytes in type I AIP patients demonstrated the critical role of these immune mediators in disease pathogenesis [77,78,79]. Patients with AIP have elevated levels of serum autoantibodies against different self-antigens, such as carbonic anhydrase (CA) II [80,81], lactoferrin [82], amylase alpha2A [83], type IV collagen [84], heat-shock protein and plasminogen-binding protein [85]. These autoantibodies, along with IgG4, serve as diagnostic markers for AIP. An increased number of peripheral T-regs has also been shown to influence IgG4 antibodies in AIP [86]. The Th2 type immune response and T-regs have a critical role in type I AIP. Th2 response in AIP is primarily composed of B-cell differentiation and production of autoantibodies. Production of IgG4 and autoantibodies is further influenced by increased IL-10 secretion from T-regs, further enhancing AIP pathogenesis [87]. Furthermore, factors regulating cellular immune function, genetic polymorphism in the inhibitory receptor, as well as cytotoxic T-lymphocyte antigen 4 (CTLA4) also regulate AIP. CTLA4 polymorphism is associated with an enhanced risk of AIP relapse in patients and influences serum CTLA4 levels. Altogether, this study shows that genetic polymorphism in CTLA4 and its serum levels correlates positively in AIP patients.

Hereditary pancreatitis patients have 40% higher chances of progression to pancreatic cancer (PC) throughout their lifetime [34]. The major cause of hereditary pancreatitis is the mutation in cationic trypsinogen (*PRSS1*), serine protease inhibitor Kazal 1 (*SPINK1*) and cystic fibrosis transmembrane conductance regulator (*CFTR*) genes [88]. These genes, in one way or the other, are involved in trypsinogen activation or trypsin inhibition, which are directly or indirectly involved in pancreatic acinar cell injury [89]. These genes play a significant role in progressing the disease from AP to CP. Along with immunological factors, such as infiltrating CD4^+^ and CD8^+^ T lymphocytes, polymorphism in the major histocompatibility complex (MHC) has also been shown to play a significant role in CP pathogenesis [90,91,92,93,94]. Compared to the healthy controls, the HLADRB1*0401 allele, encoding unique motif ^70^QKRAA^74^, is significantly expressed in CP patients. A study showed that expression of the HLADRB1*0401 allele, along with other hereditary (*PRSS1, SPINK1, CFTR*) mutations and environmental factors such as chronic smoke exposure and alcohol abuse, could further predispose patients towards CP [95]. Further, pancreatic immune cell profiling revealed the differences between hereditary and idiopathic CP. In the case of idiopathic CP, there was an increased frequency of CD68^+^ macrophages, whereas CD3^+^ T-cells were significantly high in hereditary CP, suggesting that the difference between innate and adaptive immune responses in hereditary and idiopathic CP indicates differential disease pathologies [96]. Gallstones are also among the major causes of acute pancreatitis. Gallstone-mediated pancreatic duct obstruction leads to pancreatic injury, resulting in gallstone-induced AP. Initial pathophysiological events in gallstone-induced pancreatitis affect acinar cells, causing strong acute phase response. A genetic predisposition for gallstone formation also increases the risk of AP episodes [97,98].

## 5. Impact of Targeting the Adaptive Immune Arm in Pancreatitis

**Figure 3:**
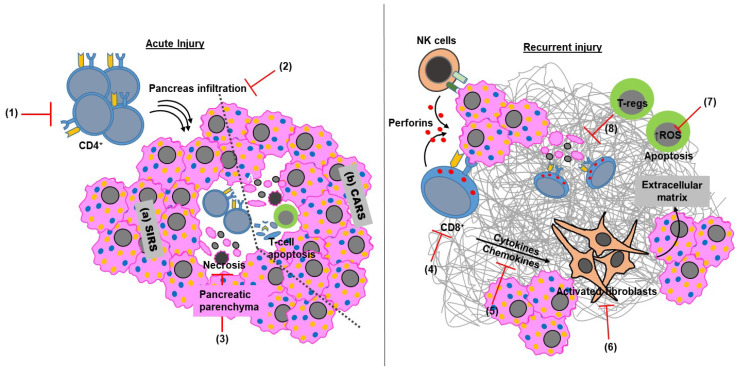
Targeting adaptive immune mediators during acute and chronic pancreatitis. Genetic ablation using athymic or T-lymphocyte-deficient mice (1) or reduced infiltration (2) of T-lymphocytes decreases the severity of acute pancreatitis. Therefore, targeting infiltrating T-lymphocytes (2) and tissue necrosis (3) is a promising strategy to reduce the acute phase responses like (a) SIRS or (b) CARS in AP. Recurrent pancreatic injury causes irreversible damage, which is effectively curtailed by targeting cytotoxic mediators (4 and 5). Targeting the inflammatory stimulus (6) by these adaptive and innate immune cells reduces pancreatic stellate cell activation (7), leading to the maintenance of pancreatic parenchyma and decreased ECM deposition. Reducing the T-reg apoptosis (8) by targeting intracellular ROS is also a promising strategy in reducing acinar cell apoptosis (9), inflammation and the maintenance of pancreatic parenchyma. SIRS; systemic immunological response syndrome. CARS; compensatory anti-inflammatory response syndrome.

As the primary event during the AP episode is the robust inflammatory response, therapeutic targeting using anti-inflammatory agents remains an area of interest. Various anti-inflammatory agents, such as Sivelestat, Glycyrrhizine, Rofecoxib, Flavocoxid and Lisinopril, have been studied in AP therapy (Figure 3). The detailed mechanisms of action targeting both the innate and adaptive immune arms by these agents in AP have been discussed elsewhere [99]. Among different anti-inflammatory agents, IL-6 inhibitors have showed promise towards AP therapy. The IL-6 inhibitor Tocilizumab, used as an anti-inflammatory agent in different inflammatory disorders, has been shown to alter the severity of AP in animal models. Major contributing factors in CP pathology are pancreatic tissue fibrosis and abundantly infiltrated T-lymphocytes. Targeting T-lymphocytes has proven to reduce the severity of disease in animal models [14]. Similarly, macrophages and PSCs are the predominant inducers of fibrosis in CP. Alternatively, activated macrophages interact with PSCs by IL-4/IL-13 signaling, and pharmacological targeting of IL4/IL13 using blocking peptides showed reduced fibrosis in CP animal models [13]. Using natural products, such as apigenin, rhein and tocotrienol-rich fraction (TRF) from palm oil, for targeting PSCs shows promise in reversing fibrosis in CP [100,101,102]. Some reports demonstrate that recovering T-reg loss and restoring IL-10 levels during CP suppress CD8^+^ T-cell over-activation and hence reduces the severity of CP [39,86]. Apart from targeting, the major area of focus in pancreatitis research at present is finding a robust, reliable and promising diagnostic marker for determining the severity of AP and MOF driven by AP. The systemic alteration of CD4^+^ T-lymphocyte and B-lymphocytes during the early phase of AP, indicating persistent MOF [29], and increased circulating levels of CD4^+^ T-cell cytokines such as IL22 [55], TGFβ and IL10 during acute and chronic pancreatitis, establishes the utility of adaptive immune effectors as the predictors of disease severity in the context of different etiological contributors. ONe study has demonstrated the significant differences between the type of circulating immune cells in case of RAP, CP and pancreatic cancer. This retrospective analysis of human serum samples from control, RAP, CP and PC patients showed a unique immune signature that can serve as a novel diagnostic marker as well as a disease predictor in various pancreatic pathologies [103]. A recent study by Zhang et al. utilized innate and adaptive immune cells as a predictor of disease severity. The study showed a significant increase in peripheral CD14^hi+^CD16^−^ monocytes within 48 h of AP onset. This increase in peripheral CD14^hi+^CD16^−^ monocytes was followed by a concomitant reduction in HLA-DR expression, as well as CD4^+^T-cells. This difference was more profound in the case of SAP; therefore, both of these factors in combination can be used as a predictor of AP severity [104].

## 6. Conclusions

The clinical progression of pancreatitis depends on both the extent of pancreatic tissue damage and the degree of the systemic immune response. Secondary factors, such as infections or vascular permeability, are also implicated in sepsis or MOF during pancreatic insult. Typically, the infiltration of immune cells is a protective response generated by the immune system to curtail the pancreatic damage, but as the disease progresses, the overwhelming anti-inflammatory response leads to a systemic immunosuppressive environment, leading to the onset of secondary complications. The adaptive immune arm plays a significant role in this entire cascade. During the initial episodes, high infiltration of T-(CD4^+^ and CD8^+^) and B-cells facilitate the recovery from acinar cell damage by clearing the apoptotic cells, reducing the inflammation and restoring the immunosuppressive environment. The situation becomes complicated when the onset of AP leads to immune syndromes such as SIRS and CARS, resulting in fatal outcomes. During CP, CD8^+^ and NKT-cells are the major players inducing cell-mediated cytotoxicity. Their exclusive role in CP can be further supported by their abundance in the fibrotic tissue. It will be interesting to know how adaptive immune cell repertoire changes with different therapeutic interventions and how it helps in resolving the onset as well as the severity of the disease.
